# Supplementary material for: The impacts of ocean acidification on marine trace gases and the implications for atmospheric chemistry and climate
Source: Proc Math Phys Eng Sci. 2020 May 13;476(2237):20190769. doi: 10.1098/rspa.2019.0769 (PMC7277135; doi:10.1098/rspa.2019.0769)
Supplement: Supplementary Information [file rspa20190769supp1.pdf]

Supplementary Information for: The impacts of ocean acidification on marine trace gases and the implications for atmospheric chemistry and climate

Frances E. Hopkins[1], Parvadha Suntharalingam[2], Marion Gehlen[3], Oliver Andrews[4], Stephen D. Archer[5], Laurent Bopp[6,7], Erik Buitenhuis[2], Isabelle Dadou[8], Robert Duce[9], Nadine Goris[10], Tim Jickells[2], Martin Johnson[2], Fiona Keng[11,12], Cliff S. Law[13,14], Kitack Lee[15], Peter S. Liss[2], Martine Lizotte[16], Gillian Malin[2], J. Colin Murrell[2], Hema Naik[17], Andrew P. Rees[1], Jörg Schwinger[10], Philip Williamson[2]

[1] Plymouth Marine Laboratory, Prospect Place, Plymouth, UK

[2] School of Environmental Sciences, University of East Anglia, Norwich Research Park, Norwich, NR4 7TJ, UK

[3] Laboratoire des Sciences du Climat et de l'Environnement, Institut Pierre Simon Laplace, Orme des Merisiers, Gif-sur-Yvette cedex, France

[4] School of Geographical Sciences, University of Bristol, University Road, Bristol, BS8 1SS

[5] Bigelow Laboratory for Ocean Sciences, Maine, USA

[6] Laboratoire de Météorologie Dynamique, Institut Pierre-Simon Laplace, CNRS-ENS-UPMC-X, Département de Géosciences, Ecole Normale Supérieure, France

[7] Université Ecole Polytechnique, Sorbonne Université, Paris, France.

[8] Laboratoire d'Etudes en Géophysique et Oceanographie Spatiales, University of Toulouse, France

[9] Departments of Oceanography and Atmospheric Sciences, Texas A&M University, College Station, Texas, USA

[10] NORCE Climate, Bjerknes Centre for Climate Research, Bergen Norway

[11] Institute of Ocean and Earth Sciences (IOES), University of Malaya, Kuala Lumpur, Malaysia

[12] Institute of Graduate Studies (IGS), University of Malaya, Kuala Lumpur, Malaysia

[13] National Institute of Water and Atmospheric Research, Wellington, New Zealand

[14] Department of Chemistry, University of Otago, Dunedin, New Zealand

[15] Division of Environmental Science and Engineering, Pohang University of Science and Technology, Pohang, South Korea.

[16] Department of Biology, Université Laval, Quebec City, Canada

[17] CSIR-National Institute of Oceanography, Dona Paula 403004, Goa, India

Author for correspondence: Frances E. Hopkins email: fhop@pml.ac.uk

Table S1: Response of DMS and/or DMSP to high CO_2 in unialgal culture experiments.

Study	Species	Strain	Response to high CO_2	
Avgoustidi et al. 2012	*E. huxleyi*	CCMP1516 (non-calc)	[DMS]	↓
Spielmeyer & Pohnert 2012	*T. pseudonana*<br>*P. tricornutum*	CCMP1335<br>CCMP2561	[DMSP] +T<br>[DMSP] +CO_2	↓<br>↓
	E. huxleyi	RCC1242 (non-calc)<br>RCC1731 (calc)	[DMSP] +T<br>[DMSP] +CO_2	↑<br>↑
Arnold et al. 2013	*E. huxleyi*	CCMP373 (non-calc)	[DMS] +CO_2<br>[DMS] +T<br>[DMS]+TCO_2	↓<br>↑<br>↑
Webb et al. 2015	*E. huxleyi*	RCC1229 (calc)	[DMS] [DMSP]<br>DMSP/cell vol.	↔<br>↓
Olson et al. 2017	*E. huxleyi*	CCMP374 (non-calc)<br>CCMP 2668 (calc)	DMSP/cell vol.<br>DMSP/cell vol.	↔<br>↓

non-calc = non calcifying, calc = calcifying

Table S2. Summary of the results of nine mesocosm studies that have reported the response of DMS to high CO_2

Study	Location	Month (Season)	pCO_2 levels	DMS response to high CO_2 (change from 350µatm) (%)	Integrated DMS (350-750µatm) (nmol L^{-1})	Calculation Basis
Avgoustidi et al. 2012	Bergen, Norway	May (Spring)	300:700	-38%	16.7: 10.4	2 treatment (2 replicates)
Vogt et al. 2008	Bergen, Norway	May (Spring)	300:690	26%	9.3 - 11.7	2 treatments (3 replicates)
Hopkins et al. 2010	Bergen, Norway	May (Spring)	320:760	-40%	8.8 - 5.3	2 treatments (3 replicates)
Webb et al. 2015	Bergen, Norway	May (Spring)	350 - 750	-22%	1.4 - 1.1	Linear fit to pCO2 vs integrated DMS (n=9)
Kim et al. 2010	Korea	Not reported	360 - 730	18%	3.3 - 5.2	2 treatments (3 replicates)
Park et al. 2014	Korea	May (Spring)	350 - 750	-42%	27.4 - 15.8	Linear fit to pCO2 vs integrated DMS (n=6)
Archer et al. 2013	Svalbard	June (Summer)	350 - 750	-13%	7.5 - 8.7	Linear fit to pCO2 vs integrated DMS (n=9)
Webb et al. 2016	Baltic Sea	June (Summer)	350 - 750	-9%	4.3 - 3.9	Linear fit to pCO2 vs integrated DMS (n=9)
Archer et al. 2018	Canary Islands	October (Autumn)	400 - 800	-20%	5.5 - 4.5	Linear fit to pCO2 vs integrated DMS (n=9)

Table S3: Summary of mesocosm studies investigating the effect of increased pCO_2 on the halocarbon emissions in three different locations.

**Study**	**Hopkins et al. 2010**	**Hopkins et al. 2013**	**Webb et al. 2016**
**Location**	Bergen, Norway	Kongsfjorden, Svalbard	Tvärminne Storfjärden, Baltic Sea
**Character**	Temperate coastal	Polar	Brackish
**pCO_2 levels (µatm)**	380, 750	185 - 1420	600 - 1650
**Date**	May 2006	June/July 2010	June 2012
**Dominant biological community**	Picoeukaryotes	Nanoeukaryotes Picoeukaryotes	Chlorophytes Cryptophytes
**General biological response to high pCO_2 levels**	Reduced under high pCO_2	Increased under high pCO_2 upon nutrient enrichment	Unclear
**Bromocarbon response**	Insignificant	No clear effect	Insignificant
**Iodocarbon response**	Unclear	CH_3 reduced with increasing pCO_2	Insignificant
